# A Prediction Rule to Stratify Mortality Risk of Patients with Pulmonary Tuberculosis

**DOI:** 10.1371/journal.pone.0162797

**Published:** 2016-09-16

**Authors:** Helder Novais Bastos, Nuno S. Osório, António Gil Castro, Angélica Ramos, Teresa Carvalho, Leonor Meira, David Araújo, Leonor Almeida, Rita Boaventura, Patrícia Fragata, Catarina Chaves, Patrício Costa, Miguel Portela, Ivo Ferreira, Sara Pinto Magalhães, Fernando Rodrigues, Rui Sarmento-Castro, Raquel Duarte, João Tiago Guimarães, Margarida Saraiva

**Affiliations:** 1 Department of Pneumology, Centro Hospitalar São João, Porto, Portugal; 2 Life and Health Sciences Research Institute (ICVS), School of Health Sciences, University of Minho, Braga, Portugal; 3 ICVS/3B’s - PT Government Associate Laboratory, Braga/Guimarães, Portugal; 4 Department of Clinical Pathology, Centro Hospitalar São João, Porto, Portugal; 5 Institute of Public Health, University of Porto, Porto, Portugal; 6 Department of Internal Medicine, Hospital do Santo Espírito da Ilha Terceira, Angra do Heroísmo, Portugal; 7 Faculty of Psychology and Educational Sciences, University of Porto, Porto, Portugal; 8 School of Economics and Management, University of Minho, Braga, Portugal; 9 Department of Radiology, Centro Hospitalar do Porto, Porto, Portugal; 10 Department of Infectious Diseases, Centro Hospitalar do Porto, Hospital Joaquim Urbano Unit, Porto, Portugal; 11 Department of Pneumology, Centro Hospitalar de Vila Nova de Gaia/Espinho, Vila Nova de Gaia, Portugal; 12 Referral Centre for Multidrug-resistant tuberculosis in the Northern Region of Portugal, Chest Disease Centre of Vila Nova de Gaia, Vila Nova de Gaia, Portugal; 13 General Directorate of Health, Lisboa, Portugal; 14 Department of Biochemistry, Faculty of Medicine, University of Porto, Porto, Portugal; 15 i3S - Instituto de Investigação e Inovação em Saúde, University of Porto, Portugal; 16 IBMC - Instituto de Biologia Molecular e Celular, University of Porto, Porto, Portugal; University of Otago, NEW ZEALAND

## Abstract

Tuberculosis imposes high human and economic tolls, including in Europe. This study was conducted to develop a severity assessment tool for stratifying mortality risk in pulmonary tuberculosis (PTB) patients. A derivation cohort of 681 PTB cases was retrospectively reviewed to generate a model based on multiple logistic regression analysis of prognostic variables with 6-month mortality as the outcome measure. A clinical scoring system was developed and tested against a validation cohort of 103 patients. Five risk features were selected for the prediction model: hypoxemic respiratory failure (OR 4.7, 95% CI 2.8–7.9), age ≥50 years (OR 2.9, 95% CI 1.7–4.8), bilateral lung involvement (OR 2.5, 95% CI 1.4–4.4), ≥1 significant comorbidity—HIV infection, diabetes mellitus, liver failure or cirrhosis, congestive heart failure and chronic respiratory disease–(OR 2.3, 95% CI 1.3–3.8), and hemoglobin <12 g/dL (OR 1.8, 95% CI 1.1–3.1). A tuberculosis risk assessment tool (TReAT) was developed, stratifying patients with low (score ≤2), moderate (score 3–5) and high (score ≥6) mortality risk. The mortality associated with each group was 2.9%, 22.9% and 53.9%, respectively. The model performed equally well in the validation cohort. We provide a new, easy-to-use clinical scoring system to identify PTB patients with high-mortality risk in settings with good healthcare access, helping clinicians to decide which patients are in need of closer medical care during treatment.

## Introduction

Tuberculosis (TB) remains a major global health problem, with an estimated 9.6 million new cases and 1.5 million deaths in 2014 [1]. In Portugal, the incidence was still 25/100.000 inhabitants (intermediate incidence rate) and, contrasting to the majority of other European countries, most of the new TB cases are Portuguese native. A very recent report [2] analysed the social profile of the highest TB incidence areas in Portugal between 2002 and 2012 and concluded that immigrants comprised only 1.6 to 1.8% in the region of Porto, while the highest proportion was seen in the Lisbon area (8.4–8.8%), where larger migration influx has occurred mainly from sub-Saharan African former colonies. In spite of the greater incidence as compared to other countries, treatment success rate in Portugal is high [1] and the case-fatality rate has been below the European Union average [3], which accounts for the efficiency of the national healthcare system.

An increased risk of death from TB has been attributed to drug resistance acquisition and HIV coinfection, especially in developing countries with high incidence [3]. However, following population-based epidemiological studies in regions of low and intermediate TB incidence, other predictors of mortality have been identified. This was the case of increasing age, male gender, the occurrence of extrapulmonary TB and several comorbidities [3–7]. Therefore, objective clinical assessment of risk factors may help lowering the death rate associated with TB by selecting those patients who might be in need of increased clinical supervision or advanced medical treatment.

The use of clinical prediction rules (CPR) gained has relevance in the field of lung diseases in the last decades [8]. Although several prediction scores have been developed in the field of TB, most of them are available for diagnostic purposes [9–13], with only three providing prognosis-centred CPRs [6, 14, 15]. Among these, none is representative of a low to intermediate incidence region, with low rates of drug resistance, in both hospital and ambulatory settings. In this context, we developed a TB risk assessment tool (TReAT) based on readily available clinical features, with the aim of stratifying the risk of death among pulmonary TB (PTB) patients and possibly helping on the decision for different management options.

## Materials and Methods

### Study design and patient population

For the derivation (training) set, the clinical records of patients with *Mycobacterium tuberculosis* positive culture at a University-affiliated hospital (Hospital São João—HSJ, Porto) during the period of 7 years (2007–2013) were retrospectively analysed. TB cases were defined according to the WHO guidelines and treatment was administered by DOT 5–7 days/week, with the recommended treatment regimens [16]. Exclusion criteria were: i) exclusively extrathoracic TB; ii) age <18 years and iii) lack of information (no registries found). Subjects were categorized according to the disease site as: i) exclusively pulmonary; ii) pleural, with or without proven PTB; or iii) combined extrathoracic and PTB. Extrathoracic involvement was defined as disease in organs other than the lungs or pleura, with either *M*. *tuberculosis* culture isolation or histologic demonstration of caseating granulomas [16].

The validation set was provided by the Chest Disease Centre (CDC) of Vila Nova de Gaia, an ambulatory referral centre for TB screening and treatment in a large urban area of the north of Portugal. Since mortality of patients diagnosed at the hospital is higher than what is observed in the non-hospital setting, we forced a ratio cases (deaths) to controls (survivors) similar to the derivation cohort, by using an entry-time-matched validation set. Cases that immediately preceded and/or immediately followed each of the fatalities occurring between 2007 and 2014 were defined as time-matched controls (survivors). No particular pairing was performed as the order of the individuals in the dataset was arbitrary.

The reporting of this study conforms to the Strengthening the Reporting of Observational Studies in Epidemiology (STROBE) statement [17] and to the CHecklist for critical Appraisal and data extraction for systematic Reviews of prediction Modelling Studies (CHARMS) [18].

### Data collection

For the derivation set, data was collected from both the HSJ clinical files and the Portuguese regional surveillance system (SVIG-TB) database. Specifically, information was collected for: age, gender and lifestyle factors (smoking status and alcohol intake); HIV infection status; pharmacological immunosuppression; active cancer; diabetes mellitus; liver failure or cirrhosis; stages 4 and 5 chronic kidney failure; congestive heart failure (CHF); and chronic respiratory disease (CRD). The definition of the coexistent illnesses is detailed in the footnotes for Table 1. Baseline clinical features available at time of diagnosis (before patients started treatment) were collected. These included: time of symptoms onset; the presence of three respiratory (cough, hemoptysis, dyspnea) and three constitutional symptoms (fever, night sweats, weight loss); hemoglobin and C-reactive protein (CRP) values; acute hypoxemic respiratory failure (defined as newly onset partial pressure of oxygen decrease to <60 mmHg, or arterial oxygen saturation <90%); and digital images of plain chest radiographs. Baseline chest radiographs were blindly analysed by two independent physicians according to the lesions extent, the presence of lung cavitation and pleural effusion. Disagreement between readers was resolved through a consensus read by a third physician. Most cases had microscopic examination of auramine-stained sputum slides, ranking acid-fast bacilli load as negative, 1+, 2+, or 3+ [19] and a drug susceptibility profile was also available. Deaths that occurred during the first 6 months after diagnosis were classified as TB death [16]. The survival time was calculated between dates of the first microbiological sampling (which allowed for the provisional diagnosis before culture positivity could be ascertained) and death. Patients were censored at the date of the last visit if they were lost to follow-up or at the end of TB treatment. Data were recorded as missing if information could not be ascertained by review of paper and electronic charts. Missing values within the derivation set are detailed on Table 1. For the validation set, only cases with complete data for the predictors included in the clinical score were considered.

**Table 1:** Study population characteristics and comparison between survivor and fatality groups. Continuous variables are presented as mean±SD or median (25th–75th percentile). The proportions in this table reflect the number of patients with each finding divided by the total number of patients for whom data were available.

Clinical feature		All (n = 681)	Survivors (n = 560)	Fatalities (n = 121)	*P* value
Age years, median (IQR)		47 (35–64.5)	45 (33–59)	63 (46.5–76.5)	<0.001^a^
Male gender, n (%)		501/681 (73.6)	405/560 (72.3)	96/121 (79.3)	0.112
Former or current smoker, n (%)		341/555 (61.4)	274/470 (58.3)	67/85 (78.8)	<0.001^a^
Comorbidities^b^, n (%)	Alcohol abuse	157/602 (26.1)	123/512 (24)	34/90 (37.8)	0.006^a^
	HIV positive	117/615 (19)	91/517 (17.6)	26/98 (26.5)	0.039^a^
	Immunosuppression	42/673 (6.2)	34/553 (6.1)	8/120 (6.7)	0.831
	Malignancy	43/672 (6.4)	14/553 (2.5)	29/119 (24.4)	<0.001^a^
	Diabetes mellitus	84/677 (12.4)	59/557 (10.6)	25/120 (20.8)	0.002^a^
	Liver failure or cirrhosis	91/669 (13.6)	67/549 (12.2)	24/120 (20)	0.024^a^
	Chronic kidney disease^c^	35/676 (5.2)	25/557 (4.5)	10/119 (8.4)	0.080
	Congestive heart failure	52/666 (7.8)	32/549 (5.8)	20/117 (17.1)	<0.001^a^
	Chronic respiratory disease	109/660 (16.5)	74/544 (13.6)	35/116 (30.2)	<0.001^a^
TB site, n (%)	Pulmonary	478/681 (70.2)	393/560 (70.2)	85/121 (70.2)	0.939
	Pleural ± pulmonary	90/681 (13.2)	75/560 (13.4)	15/121 (12.4)	
	Pulmonary + extrathoracic	113/681 (16.6)	92/560 (16.4)	21/121 (17.4)	
Time of symptoms (weeks), median (IQR)		7 (4–12)	8 (4–13)	4 (2.8–11)	0.002^a^
Main symptoms,n(%)	Cough	450/577 (78)	374/477 (78.4)	76/100 (76)	0.597
	Hemoptysis	99/571 (17.3)	90/475 (18.9)	9/96 (9.4)	0.024^a^
	Dyspnea	240/575 (41.7)	173/472 (36.7)	67/103 (65)	<0.001^a^
	Fever	345/578 (59.7)	284/479 (59.3)	61/99 (61.6)	0.668
	Night sweats	225/490 (45.9)	195/415 (47)	30/75 (40)	0.264
	Weight loss	335/539 (62.2)	271/448 (60.5)	64/91 (70.3)	0.078
Bacillary load^d^, n (%)	0	138/477 (28.9)	114/384 (29.7)	24/93 (25.8)	0.823
	1+	64/477 (13.4)	52/384 (13.5)	12/93 (12.9)	
	2+	99/477 (20.8)	80/384 (20.8)	19/93 (20.4)	
	3+	176/477 (36.9)	138/384 (35.9)	38/93 (40.9)	
Drug resistance,n(%)	Isoniazide^e^	30/655 (4.6)	23/545 (4.2)	7/110 (6.4)	0.327
	Riphampicin	6/655 (0.9)	5/545 (0.9)	1/110 (0.9)	0.993
	Pyrazinamide^f^	6/294 (2)	5/248 (2)	1/46 (2.2)	0.945
	Ethambutol	7/655 (1.1)	5/545 (0.9)	2/110 (1.8)	0.402
Hypoxemic respiratory failure, n (%)		115/595 (19.3)	64/491 (13)	51/104 (49)	<0.001^a^
Hemoglobin g/dL, mean ±SD		12.0 ± 2.2	12.2 ± 2.1	11.0 ± 2.2	<0.001^a^
CRP mg/L, median (IQR)		79.4 (32.3–126.9)	74.9 (29.2–125.4)	90.5 (42.5–144)	0.011^a^
Cavitation, n (%)		265/613 (43.4)	219/494 (44.3)	47/119 (39.5)	0.339
Bilateral lung involvement, n (%)		336/598 (56.2)	248/484 (51.2)	88/114 (77.2)	<0.001^a^
Pleural effusion, n (%)		148/605 (24.5)	110/489 (22.5)	38/116 (32.8)	0.021^a^

^a^Statistically significant results.

^b^HIV infection = positive titer of antibodies to HIV; Immunosuppression = organ transplant and patients receiving the equivalent of ≥15 mg/day of prednisolone for ≥1 month, other immunosuppressive drugs, or TNF-α antagonists; Active cancer = any cancer except basal- or squamous cell cancer of the skin, that was active at the time of presentation; Diabetes mellitus = history of diabetes or fasting blood glucose concentration ≥126 mg/dL at 2 different time points; Liver failure/cirrhosis = chronic liver disease with coagulopathy and hypoalbuminaemia or a clinical or histologic diagnosis of cirrhosis; Chronic Kidney Disease = history of chronic renal disease or abnormal blood urea nitrogen and creatinine concentrations documented in the medical record; Congestive heart failure = systolic or diastolic ventricular dysfunction documented by history, physical examination, chest radiograph and/or echocardiogram; Chronic respiratory disease = COPD and structural lung disease.

^c^CKD stages 4 or 5.

^d^Only cases of culture confirmation on sputum (the remaining subjects were diagnosed through gastric aspirate, bronchial wash, bronchoalveolar lavage, pleural fluid or biopsy cultures).

^e^Missingness of 3.8% due to contaminated culture or non-representative sampling.

^f^Pyrazinamide resistance was not routinely assessed until May 2011.

CRP—C-reactive protein; IQR—interquartile range; HIV—human immunodeficiency virus; SD—standard deviation; TB—tuberculosis

### Statistical analysis

Univariate analyses were conducted for all variables comparing survivors and fatalities in the derivation cohort. Continuous variables were recategorized into binary factors. In the absence of previously described thresholds in the literature, we used the Youden index criterion to estimate the optimal cut-point when giving equal weight to sensitivity and specificity [20]. For categorical variables, comparisons were made using a Chi-square test or Fisher exact test as appropriate. For continuous variables, comparisons were made using an independent group *t*-test, or a Mann-Whitney *U*-test for non-normally distributed variables.

Models to predict death in PTB patients were derived using stepwise logistic regression with 6-month mortality as the outcome measure. Eight clinically plausible interactions were tested (listed in S1 Table). The results of significant predictors were reported as odds ratios (ORs) and 95% confidence intervals (CI). Models were assessed for goodness-of-fit using receiving operator characteristic (ROC) curves and the Hosmer-Lemeshow test. Then, using the Heckman's selection model, values of significant variables with missing data were modeled and incorporated into the initial model to assess and correct for potential bias. This method attempts to control for the effect of nonrandom selection by incorporating both the observed and unobserved factors that affect nonresponse [21].

To derive a simple-to-compute risk score, the regression coefficients of the predictors were divided by the smallest coefficient and then rounded to the nearest integer [22]. For each patient, a total risk score was obtained by calculating the sum of individual points attributed to each of the variable.

Three methods were used to validate the CPR. Pearson's Chi-square tests for independence were performed to test the association between risk score groups and observed deaths on derivation and validation cohorts and for total sample. Association between categories was evaluated based on the adjusted residual scores, where absolute values >1.96 represent significant differences for 95% confidence level (*P*<0.05). Significant positive scores reveal a tendency to observe death in the considered group. To describe the accuracy of the model for predicting mortality, we reported the sensitivity, specificity and test predictive values. The area under the ROC curve (AUC) and its 95% confidence interval (CI) were determined and compared in the derivation and validation cohorts. All the statistical analyses were performed using the SPSS software program, version 22 (IBM^®^ SPSS^®^, Inc.) and STATA, version 14 (STATA Corp) for Heckman modeling.

### Ethics

The study protocol was approved by the Health Ethics Committees of the HSJ (approval number 109–11), the North Health Region Administration (approval number 71–2014) and the Portuguese Data Protection Authority (approval number 12174–2011). The requirement to obtain informed written consent from each individual was waived, as the study was limited to the review of existing medical records. To ensure confidentiality, each case was anonymized by the assignment of a random identification number.

## Results

### Study design

**Fig 1:**
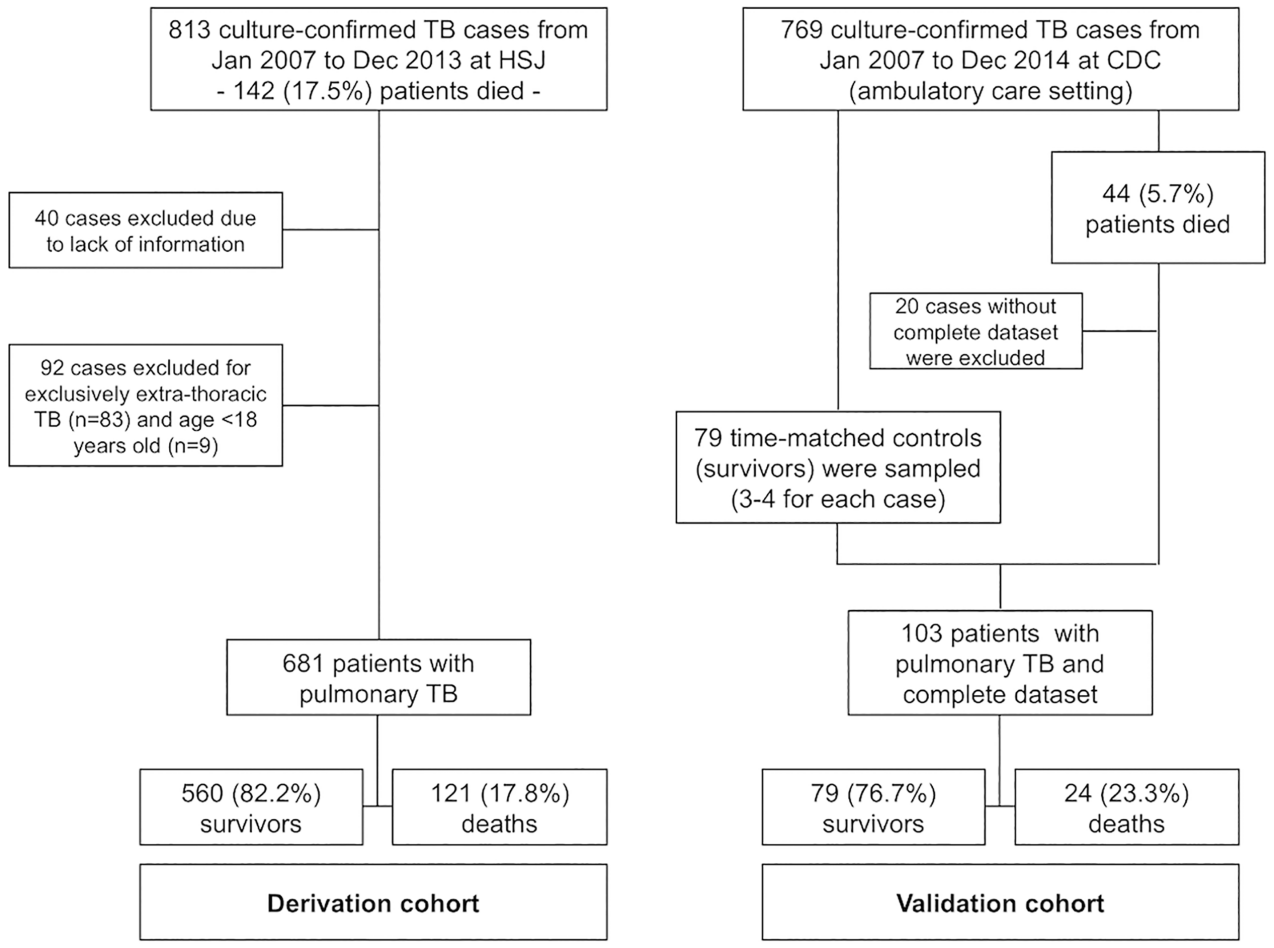
Flow chart for the selection of the participating patients, according to the STROBE guidelines. HSJ—Hospital São João, Porto, Portugal; CDC—Chest Disease Centre (ambulatory care), Vila Nova de Gaia, Portugal.

Between 2007 and 2013, 813 culture-confirmed new TB cases were diagnosed at the HSJ (both inpatient and outpatient), of which 142 (17.5%) were reported to have died within 6 months of diagnosis. Patients with overall lack of information (n = 40), with exclusively extrathoracic TB (n = 83) or <18 years (n = 9) were excluded from the study (Fig 1). A total of 681 patients were included for univariate analysis, including 121 (17.8%) fatalities within 6 months (183 days) of diagnosis, with median survival time of 33 days (range 1 to 182). Of the 560 not known to have died, 60 were lost to follow-up after a median of 138.5 days (range 6 to 182) and the remaining 500 were censored at the end of follow-up period. The vast majority of cases included in the study were Portuguese-born caucasians (96.5%) and only 0.4% of cases harboured multidrug-resistant strains.

The validation cohort was from an ambulatory referral centre (CDC), which followed 769 patients between 2007 and 2014 and registered a mortality rate of 5.7%. Of the 44 CDC registered deaths, only 24 patients had complete data. Seventy-nine survivors were included to match these fatalities, leading to a validation cohort of 103 patients (Fig 1), in a 3:1 ratio (except for 7 fatal cases, which had 4 matching controls).

### Development of a practical CPR to assess risk of death

The general characteristics of the study cohort and specific associations with death are presented in Table 1. The significant variables were then tested in multivariate logistic regression model with 6-month mortality as the dependent variable. Participants were categorized by age groups, according to a threshold based on Youden index criterion, which defined an optimal cut-point of 53.5 years (50 years, if rounded to nearest multiple of 10). Hemoglobin <12 g/dL defined anemia in both genders. Self-reporting variables with more than 20% of missing values were excluded, as the validity of these data is doubtful. This was the case for smoking, alcohol habits and time of symptoms. CRD and liver failure/cirrhosis were collinear with smoking and alcohol abuse, respectively, and worked as surrogates for those exposures. Based on clinical reasoning, malignancy was also excluded, as it related to patients with incurable active cancer and it was strongly associated with mortality itself. Six interactions between variables were identified, but the resulting ORs were always similar or even lower than with significant variables alone (S1 Table). In addition to increased complexity, there was no benefit in terms of model performance to predict risk when these interactions were included. Hence, they were omitted from the model. Using backward selection [23], a final parsimonious model with 5 predictors (Table 2, S1 Fig) was generated.

**Table 2:** Multivariable logistic regression analysis for deriving tuberculosis risk score for death.

Predictor	Crude OR (95% CI)	Regression coefficient	Multivariable OR (95% CI)	Weight for risk score
Hypoxemic respiratory failure	6.7 (4.2–10.9)	1.543	4.7 (2.8–7.9)	3
Age ≥50 years old	4.2 (2.6–6.8)	1.050	2.9 (1.7–4.8)	2
Bilateral lung involvement	3.4 (2.0–5.8)	0.899	2.5 (1.4–4.4)	1
At least 1 significant comorbidity^a^	3.4 (2.1–5.4)	0.813	2.3 (1.3–3.8)	1
Hemoglobin <12 g/dL	2.5 (1.6–4.0)	0.600	1.8 (1.1–3.1)	1

^a^ At least one of these comorbidities: HIV infection, diabetes mellitus, liver failure or cirrhosis, congestive heart failure and chronic respiratory disease.

CI—confidence interval; OR—odds ratio

The equation of the prediction model and Heckman’s selection equation were not independent (χ^2^_(1)_ = 5.12, *P* = 0.023), an argument that justifies the need to apply this procedure. The marginal effects for each of the predicting variables were compared between both models (S2 Table). The maximum absolute difference found between the two models was 5% (e.g. for patients with age ≥50 years the probability of death within 6 months of diagnosis was 24% with CPR model and 29% with Heckman’s model). However, the CPR model correctly identified the marginal effect associated to each predictor (e. g. when binary variable hypoxemic respiratory failure changes from 0 to 1, the death probability changes 0.23 for CPR and 0.22 with Heckman’s model). Thus, the use of this model showed that missing information in the univariate significant variables had little or no effect on mortality risk assessment.

**Fig 2:**
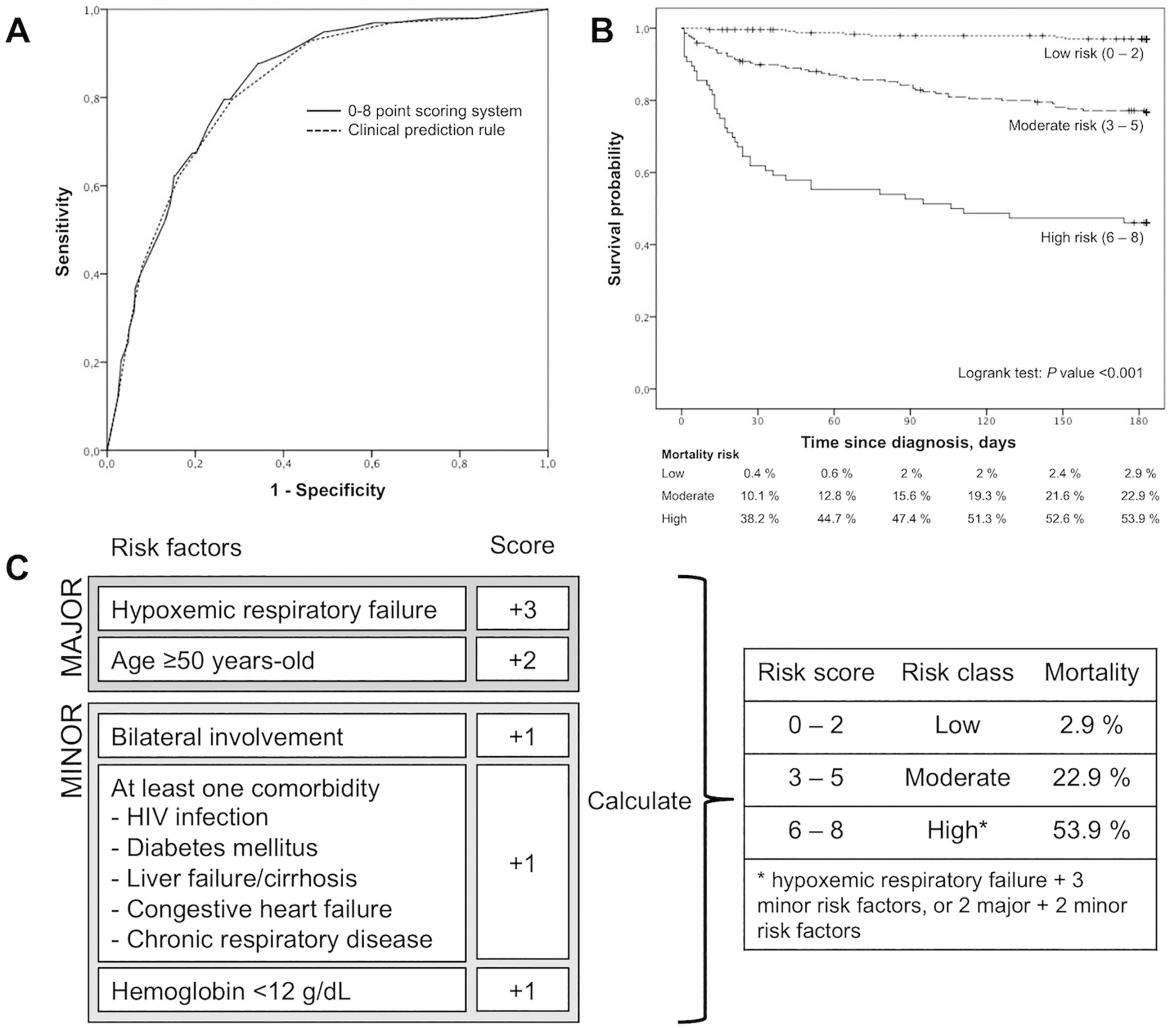
A—ROC curve for the logistic regression model (clinical prediction rule equation, S1 Fig) and clinical scoring system (0 to 8 points) | B—Kaplan-Meier estimates of survival in low-risk (clinical score 0–2), moderate-risk (clinical score 3–5) and high-risk (clinical score 6–8) tuberculosis patients. The mortality in each group at different time-points is shown below | C—TB risk assessment tool (TReAT), using baseline clinical features for stratifying patients with pulmonary tuberculosis into severity groups according to the probability of death at 6 months.

A weight for risk score was calculated for each variable, as describe above (Table 2). The strongest predictors (major criteria) of mortality were hypoxemic respiratory failure, followed by age ≥50 years. Minor criteria were bilateral lung involvement, the presence of at least one of the significant comorbidities (HIV infection, diabetes, liver failure/cirrhosis, CHF, or CRD) and hemoglobin level <12 g/dL. The accuracy of the model was then tested. A ROC curve was generated (Fig 2A), showing that the overall sensitivity and specificity of the clinical scoring system is similar to the logistic regression model.

### Development of TReAT: a scoring system to stratify the risk of death in TB patients

Based on the aforementioned clinical features and using the weights given in Table 2, a scoring system was constructed to stratify the risk of death among PTB patients using the 2 major risk factors and the 3 minor ones, and assigning a score from 0 to +8 to each patient (Fig 2B and 2C). The sensitivities, specificities and predictive values of different score values are given in Table 3. The high sensitivity and negative predictive value obtained for a score below 3 points, proved that the test performed well to identify cases of reduced death probability when scoring 0 to 2. These cases were included in the low-risk group and only a few died during follow-up (Fig 2B). The most heterogeneous group was the moderate-risk one, with a gradual decline of survival during the assessed 6 months. For a cut-off score of ≥6, the specificity and positive predictive value increased significantly. Thus, most deaths were observed in the high-risk patients scoring 6 to 8.

**Table 3:** Test characteristics with different prediction scores for mortality in the derivation cohort of patients with pulmonary tuberculosis.

Score	n (%)	Sensitivity (%)	Specificity (%)	PPV (%)	NPV (%)
≥0	539 (100)	100	0	17.8	NA
≥1	466 (86.5)	98	16.1	20.6	97.3
≥2	380 (70.5)	96.9	35.4	25	98.1
≥3	294 (54.5)	92.9	54	31.0	97.1
≥4	203 (37.7)	79.6	71.7	38.4	94
≥5	133 (24.7)	62.2	83.7	45.9	90.9
≥6	76 (14.1)	41.8	92.1	53.9	87.7
≥7	49 (9.1)	27.6	95	55.1	85.5
≥8	23 (4.3)	12.2	97.5	52.2	83.3

NA—not applicable; NPV—negative predictive value; PPV—positive predictive value

### Validation of TReAT

TReAT was validated in an independent cohort (Fig 1). By performing the validation in a non-hospital centre, a more general applicability of the CPR was tested. Importantly, a significant association was found between group scores (low-, moderate- and high-risk) and real deaths in the validation cohort and with similar magnitude (P<0.001) to what was observed for the derivation set (Table 4). Furthermore, all comparisons between sets regarding mortality within each risk group were non-significant. Association between categories was also reinforced based on the adjusted residual scores, where absolute values >1.96 indicate that the number of cases in those cells are significantly larger in moderate and high-risk groups (positive relation), or smaller in the low-risk group (negative relation), than would be expected if the null hypothesis were true, with a significance level of 0.05. In support of the generated assessment tool (TReAT), there was no significant difference in the AUCs between the derivation (0.82, 95% CI 0.78–0.87) and the validation cohorts (0.84, 95% CI 0.76–0.93; *P* = 0.72).

**Table 4:** Comparison of risk groups mortality in the derivation and validation cohorts^a^.

	Derivation cohort		Validation cohort		Total sample	
	Deaths (n,%)	*ar*^b^	Deaths (n,%)	*ar*	Deaths (n,%)	*ar*
Low risk (score 0–2)	7/245 (2.9)	-8.4	2/54 (3.7)	-4.9	9/299 (3)	-9.6
Moderate risk (score 3–5)	50/218 (22.9)	2.4	13/38 (34.2)	2.0	63/256 (24.6)	2.9
High risk (score 6–8)	41/76 (53.9)	8.7	9/11 (81.8)	4.9	50/87 (57.5)	9.8
**Total**	**98/539 (18.2)**		**24/103 (23.3)**		**122/642 (19)**	

^a^Association between risk groups and real deaths for derivation cohort (χ^2^ = 107.3, P<0.001) and for validation cohort (χ^2^ = 35.2, P<0.001) were significant. The P values for the comparisons of real mortality between sets for each risk groups are as follows: low-, *P* = 0.67; moderate-, *P* = 0.15; high-, *P* = 0.11.

^b^Absolute values >1.96 represent significant differences for 95% confidence level (P<0.05). *ar—Adjusted Residual* scores

## Discussion

This study offers, to the best of our knowledge, the first CPR for TB death prognosis in a high-income region of low to intermediate TB incidence, without expressive multi-drug resistance, comprising both hospital and ambulatory settings. The aim of this CPR is to signal confirmed cases of TB who are at higher risk of death and thus need a stricter medical supervision. Other TB scoring systems have been previously developed, but a few limitations likely hinder their use in this specific context. To the best of our knowledge, only three prognostic rules have been developed for TB. Wejse et al. proposed the first prediction rule (the Bandim TBscore) in a low-resource country (Guinea-Bissau), based on five symptoms and six clinical signs [14]. There are a few reasons why this score may not be applicable to our setting: i) it was never validated in a high-income region, which has very different epidemiological features and better healthcare access; ii) a large proportion of the patients included in the Bandim TBscore study were HIV-infected and had no antiretroviral treatment available, which could independently affect mortality; and iii) in the same study more than half of the cases were smear negative and had no culture confirmation of TB diagnosis. In our cohort, smear negative cases comprised only one third of the subjects, mostly old and HIV-infected patients, but all had culture-proven TB. Another prognostic score was developed by Horita et al. to predict in-hospital death in a context similar to ours [6]. However, the analysis was biased by excluding multidrug resistant-TB and HIV-infected subjects, which are known to increase mortality [3, 4, 24, 25]. Also, other comorbidities were not included in their score and diabetes was not significantly associated to death (possibly due to the small sample size), contrarily to the majority of other studies [26–28], including the present one. By including coexistent conditions in TReAT, the weight given to age was attenuated, as young patients with diseases, like HIV/AIDS and liver failure/cirrhosis, were also considered at risk. Moreover, all patients included in the Horita et al. study were admitted to the ward, which is hardly representative for the overall population of culture-positive TB patients, since in many European countries, including Portugal, TB treatment is done largely outside the hospital setting. Finally, Valade et al. [15] assessed only 53 cases to propose a prognostic scoring system for TB patients admitted to the intensive care unit. It will be interesting, in future, to compare TReAT with each of these scores and analyse their relative performance and applicability to different settings.

In the present work, the fatal outcome was determined until 6 months after diagnosis, which is supported by several studies, including clinical trials [29–31], and contemplates the minimum duration of the standard multidrug treatment. Late mortality related to TB was previously described to stabilize after 6-months of treatment [30]. Nevertheless, patients died more frequently during the first 30 days after diagnosis, which did not appear to be related with diagnostic delay, since fatal cases usually had shorter time of symptoms compared to survivors (Table 1). Our results thus suggest that high-risk subjects are usually severely ill from the beginning and should, therefore, be strictly followed and start anti-TB drugs under close monitoring. Initial care in an intensive care or high dependency unit may be appropriate for some cases, particularly because they may have a slow response to standard treatment regimens. The mortality in the derivation cohort (17.5%) was higher than the national estimate (6.4%) [1] and that of the validation cohort (5.7%). This is in line with the described worse TB outcome in hospitalized patients [32, 33]. However, in-hospital mortality due to PTB seems underestimated, when those who require advanced respiratory support face a much higher death rate (up to 70%) than subjects with respiratory failure due to other causes [34].

One of the strengths of this study was the clear definition of our predictor variables, which are not dependent on patient’s recall or susceptible to clinicians’ subjective judgement. The proposed prediction model is based on intrinsic patients’ characteristics (age and comorbidities), disease extent (respiratory failure and one radiographic feature) and a measure of consumption and overall nutritional status (hemoglobin level). The relevance of these variables to the outcome of TB is in line with previous studies [3–7, 14, 31, 34–36].

Limitations of the current study include its retrospective nature and the fact that the validation set is relatively small. Owing to the retrospective design of the study, a significant number of missing values were found in certain variables. The causes of non-random missing information are very heterogeneous, mainly dependent on the patient status, or on the clinicians, who were of different medical specialties, acting in different settings (emergency room, ward, ICU, outpatient clinic), and may have neglected differential data. For instance, there was a 12.6% of missingness in the “hypoxemic respiratory failure” variable. It is possible that less severe patients without any signs of respiratory distress were not assessed for hypoxemia. On the other hand, patients who were admitted at the busy emergency room and who died during the first 24-48h after admission may have had a short description of his/her clinical condition or past history. Also, 66 patients (9%) of the derivation cohort were not tested for HIV. In a recent work [37] with 7683 TB cases notified in Northern Portugal between 2006 and 2012, 879 (11%) had also unknown HIV status (usually older patients or without history of addiction). We addressed the missingness issue by applying the Heckman method and showed the little impact on the validity of the prediction model. Moreover, sample collection was based in two centres with geographical proximity. It is however important to note that these centres had very different characteristics (hospital versus ambulatory) and yet the accuracy of TReAT was similar. Considering the small validation set, it will undoubtedly be of interest to test this CPR prospectively to other surroundings, in whole cohorts or in multicentric studies that would allow larger sample sizes.

The WHO has recently launched the End TB Strategy, which defined the lines towards TB elimination [38]. Among the discussed requirements was the importance of monitoring treatment outcomes. As TB incidence rates decrease with a combination of near-universal access to high-quality diagnosis and treatment and general socioeconomic development, it is expected that the TB death rate will become one of the core indicators for disease control [11]. Implementation of a CPR to identify patients who are more likely to die may prompt screening initiatives in particular risk groups and point directions for further programmatic interventions. On the other hand, the great variability of reported rates of hospital admission and lengths of stay for TB [39, 40] may reflect the uncertainty among clinicians regarding the definition of severity of illness. We expect that the proposed TReAT helps to bridge these gaps, providing more cost-effectiveness use of medical resources by selecting more appropriately patients that will need closer clinical surveillance.

## Supporting Information

S1 CHARMS CHecklist for critical Appraisal and data extraction for systematic Reviews of prediction Modelling Studies. Relevant items to extract from individual studies in a systematic review of prediction models.
(PDF)

S1 Fig Equations for the derived clinical prediction model that that estimates TB patient-specific death probability. *“e”* is the base of the natural logarithm, each of the terms “hypoxemia”, “age”, “bilateral”, “comorbidity” and “hemoglobin” are equal to 1 if, respectively, newly onset hypoxemic respiratory failure, age ≥50 years, bilateral lung involvement, at least 1 significant comorbidity present (HIV infection, diabetes mellitus, liver failure/cirrhosis, congestive heart failure, or chronic respiratory disease) and hemoglobin <12 g/dL (otherwise 0).
(PDF)

S1 STROBE Strengthening the Reporting of Observational Studies in Epidemiology checklist. Checklist of items that should be included in reports of observational studies.
(PDF)

S1 Table Clinically plausible interactions tested on univariate analysis. Eight clinically plausible interactions were tested and six were identified. However, the resulting odds ratios (ORs) were always similar or even lower than with significant variables alone. In addition to increased complexity, there was no benefit in terms of model performance to predict risk when these interactions were included. Hence, they were omitted from the final model.
(PDF)

S2 Table Comparison of the marginal effects between the Clinical Prediction Rule (CPR) and the two-stage Heckman model. The marginal effects for each of the predicting variables were compared between both models. The maximum absolute difference found between the two models was 5% (e.g. for patients with age ≥50 years the probability of death within 6 months of diagnosis was 24% with CPR model and 29% with Heckman’s model). However, the CPR model correctly identified the marginal effect associated to each predictor (e.g. when binary variable hypoxemic respiratory failure changes from 0 to 1, the death probability changes 0.23 for CPR and 0.22 with Heckman’s model). Thus, the use of this model showed that missing information in the univariate significant variables had little or no effect on mortality risk assessment.
(PDF)

S3 Table Comparison of the distribution of predictors for derivation and validation cohorts. (PDF)
