# Correction: A Prediction Rule to Stratify Mortality Risk of Patients with Pulmonary Tuberculosis

**DOI:** 10.1371/journal.pone.0242455

**Published:** 2020-11-11

**Authors:** Helder Novais Bastos, Nuno S. Osório, António Gil Castro, Angélica Ramos, Teresa Carvalho, Leonor Meira, David Araújo, Leonor Almeida, Rita Boaventura, Patrícia Fragata, Catarina Chaves, Patrício Costa, Miguel Portela, Ivo Ferreira, Sara Pinto Magalhães, Fernando Rodrigues, Rui Sarmento Castro, Raquel Duarte, João Tiago Guimarães, Margarida Saraiva

The raw data underlying the results of this paper are missing from the list of Supporting Information. The authors have provided the data as Supporting Information file S1 Data. With this correction [1], all relevant data are now provided.

## Supporting information

S1 Data Supplementary dataset. This file includes supplementary data.
(XLSX)
