# Supplementary material for: A Prediction Rule to Stratify Mortality Risk of Patients with Pulmonary Tuberculosis
Source: PLoS One. 2016 Sep 16;11(9):e0162797. doi: 10.1371/journal.pone.0162797 (PMC5026366; doi:10.1371/journal.pone.0162797)
Supplement: S1 CHARMS — Relevant items to extract from individual studies in a systematic review of prediction models. (PDF) [file pone.0162797.s001.pdf]

S1 CHARMS - Relevant items to extract from individual studies in a systematic review of prediction models

Domain	Key items	Reported on page #
**SOURCE OF DATA**	Source of data (e.g., cohort, case-control, randomized trial participants, or registry data)	p.4 (retrospective cohort)
**PARTICIPANTS**	Participant eligibility and recruitment method (e.g., consecutive participants, location, number of centers, setting, inclusion and exclusion criteria)	p.4 (inclusion criteria, number of centers, their setting and location), p.7 (study design)
	Participant description	p.7, p.17 (Table 1)
	Details of treatments received, if relevant	NA
	Study dates	p.7 (study design)
**OUTCOME(S) TO BE PREDICTED**	Definition and method for measurement of outcome	p.5 (definition of death outcome and survival time), p.5-6 (logistic regression with 6-month mortality as the outcome measure)
	Was the same outcome definition (and method for measurement) used in all patients?	Yes
	Type of outcome (e.g., single or combined endpoints)	Single
	Was the outcome assessed without knowledge of the candidate predictors (i.e., blinded)?	No. Collection of all data was performed sequentially at the same time, which included date of death or date of loss of follow-up. However, definition of the clinical prediction model (ie, selection of relevant predictors) was performed independently.
	Were candidate predictors part of the outcome (e.g., in panel or consensus diagnosis)?	No
	Time of outcome occurrence or summary of duration of follow-up	p.5 (event within 6 months)
**CANDIDATE PREDICTORS (OR INDEX TESTS)**	Number and type of predictors (e.g., demographics, patient history, physical examination, additional testing, disease characteristics)	p.4-5 (data collection), p.17 (Table 1)
	Definition and method for measurement of candidate predictors	p.4-5 (data collection), p.5 (criteria for redefining continuous variables into binary factors), p.17 (Table 1 footnote)
	Timing of predictor measurement (e.g., at patient presentation, at diagnosis, at treatment initiation)	p.5 (data collection at baseline, meaning at patient diagnosis)
	Were predictors assessed blinded for outcome, and for each other (if relevant)?	No. Collection of all data was performed sequentially at the same time, which included date of death or date of loss of follow-up. However, definition of the clinical prediction model (ie, selection of relevant predictors) was performed independently.
	Handling of predictors in the modelling (e.g., continuous, linear, non-linear transformations or categorised)	p.7-8 (development of a practical CPR to assess risk of death) and p.8-9 (development of scoring system TReAT to stratify the risk of death)
**SAMPLE SIZE**	Number of participants and number of outcomes/events	p.7 (study design) and Figure 1
	Number of outcomes/events in relation to the number of candidate [illegible]	p.7 (study design) and Figure 1

**MISSING DATA**	Number of participants with any missing value (include predictors and outcomes)	p.18 (Table 3, the sample size for both sets, n=539 and n=103, corresponds to the number of participants with any missing value)
	Number of participants with missing data for each predictor	p.17 (Table 1)
	Handling of missing data (e.g., complete-case analysis, imputation, or other methods)	p.6 (Heckman's selection model)
**MODEL DEVELOPMENT**	Modelling method (e.g., logistic, survival, neural network, or machine learning techniques)	p.5-6 (logistic regression)
	Modelling assumptions satisfied	Yes. We believe that is implicit throughout the text (see p.5-8): binary dependent variable; large sample size with >10 cases per predictor; observations are independent, without multicollinearity.
	Method for selection of predictors **for inclusion** in multivariable modelling (e.g., all candidate predictors, pre-selection based on unadjusted association with the outcome)	p.5-8 (pre-selection based on unadjusted association with the outcome)
	Method for selection of predictors **during multivariable modelling** (e.g., full model approach, backward or forward selection) and criteria used (e.g., p-value, Akaike Information Criterion)	p.5 and p.7-8 (stepwise backward selection)
	Shrinkage of predictor weights or regression coefficients (e.g., no shrinkage, uniform shrinkage, penalized estimation)	No shrinkage
**MODEL PERFORMANCE**	Calibration (calibration plot, calibration slope, Hosmer-Lemeshow test) and Discrimination (C-statistic, D-statistic, log-rank) measures with confidence intervals	p.6 (Models were assessed for goodness-of-fit using receiving operator characteristic (ROC) curves and the Hosmer-Lemeshow test)
	Classification measures (e.g., sensitivity, specificity, predictive values, net reclassification improvement) and whether a-priori cut points were used	p.8-9 and p.18 (Table 3)
**MODEL EVALUATION**	Method used for testing model performance: development dataset only (random split of data, resampling methods e.g. bootstrap or cross-validation, none) or separate external validation (e.g. temporal, geographical, different setting, different investigators)	p.9 and p.18 (Table 4 – external validation in a different setting)
	In case of poor validation, whether model was adjusted or updated (e.g., intercept recalibrated, predictor effects adjusted, or new predictors added)	Validation performed well. There was no need for any adjustment or update.
**RESULTS**	Final and other multivariable models (e.g., basic, extended, simplified) presented, including predictor weights or regression coefficients, intercept, baseline survival, model performance measures (with standard errors or confidence intervals)	p.8-9 (development of a scoring system, taking into account the weights derived from the regression coefficients; see also Table 2, p.18)
	Any alternative presentation of the final prediction models, e.g., sum score, nomogram, score chart, predictions for specific risk subgroups with performance	Figure 2C
	Comparison of the distribution of predictors (including missing data) for development and validation datasets	Supplementary table 2
**INTERPRETATION AND DISCUSSION**	Interpretation of presented models (confirmatory, i.e., model useful for practice versus exploratory, i.e., more research needed)	p.9-12
	Comparison with other studies, discussion of generalizability, strengths and limitations.	p.9-12
