# Supplementary material for: A Prediction Rule to Stratify Mortality Risk of Patients with Pulmonary Tuberculosis
Source: PLoS One. 2016 Sep 16;11(9):e0162797. doi: 10.1371/journal.pone.0162797 (PMC5026366; doi:10.1371/journal.pone.0162797)
Supplement: S1 Fig — “e” is the base of the natural logarithm, each of the terms “hypoxemia”, “age”, “bilateral”, “comorbidity” and “hemoglobin” are equal to 1 if, respectively, newly onset hypoxemic respiratory failure, age ≥50 years, bilateral lung involvement, at least 1 significant comorbidity present (HIV infection, diabetes mellitus, liver failure/cirrhosis, congestive heart failure, or chronic respiratory disease) and hemoglobin <12 g/dL (otherwise 0). (PDF) [file pone.0162797.s002.pdf]

S1 Fig.

The derived clinical prediction model is described by the following equations:

$$Probability\ of\ death = \frac{1}{(1 + e^{-\mathcal{Z}})}$$

$$\mathcal{Z} = -3.954 + 1.543 \times hypoxemia + 1.050 \times age + 0.899 \times bilateral + 0.813 \times comorbidity + 0.600 \times hemoglobin$$

where *"e"* is the base of the natural logarithm, each of the terms "hypoxemia", "age", "bilateral", "comorbidity" and "hemoglobin" are equal to 1 if, respectively, newly onset hypoxemic respiratory failure, age ≥50 years, bilateral lung involvement, at least 1 significant comorbidity present (HIV infection, diabetes mellitus, liver failure/cirrhosis, congestive heart failure, or chronic respiratory disease) and hemoglobin <12 g/dL (otherwise 0).
