# Supplementary material for: A Prediction Rule to Stratify Mortality Risk of Patients with Pulmonary Tuberculosis
Source: PLoS One. 2016 Sep 16;11(9):e0162797. doi: 10.1371/journal.pone.0162797 (PMC5026366; doi:10.1371/journal.pone.0162797)
Supplement: S1 Table — Eight clinically plausible interactions were tested and six were identified. However, the resulting odds ratios (ORs) were always similar or even lower than with significant variables alone. In addition to increased complexity, there was no benefit in terms of model performance to predict risk when these interactions were included. Hence, they were omitted from the final model. (PDF) [file pone.0162797.s004.pdf]

S1 Table.

Clinically plausible interactions tested on univariate analysis

Variable		n	Odds ratio	95% CI	P values
Age ≥50 years		681	3.81	2.48-5.86	<0.001
Gender (male)		681	0.68	0.42-1.10	0.114
At least 1 significant comorbidity[a]		678	3.33	2.18-5.10	<0.001
HIV		615	1.69	1.02-2.79	0.014
Time of symptoms, weeks		517	0.96	0.92-0.99	0.010
Main symptoms	Cough	577	0.87	0.53-1.45	0.598
	Hemoptysis	571	0.44	0.22-0.91	0.027
	Dyspnea	575	3.22	2.06-5.03	<0.001
	Fever	578	1.10	0.71-1.72	0.668
	Night sweats	490	0.75	0.46-1.24	0.265
	Weight loss	539	1.55	0.95-2.52	0.079
Hemoglobin <12 g/dL		612	2.74	1.80-4.18	<0.001
**Interaction terms**					
Age ≥50 years * Hemoglobin <12 g/dL		612	3.82	2.50-5.85	<0.001
Age ≥50 years * ≥1 significant comorbidity		678	3.79	2.51-5.70	<0.001
Age ≥50 years * HIV		615	2.68	1.22-5.89	0.014
Gender * Hemoglobin <12 g/dL		678	3.79	2.51-5.70	<0.001
Time of symptoms * Weight loss		464	0.98	0.95-1.01	0.262
Cough * Hemoptysis		566	0.35	0.15-0.83	0.017
Cough * Dyspnea		568	2.74	1.76-4.27	<0.001
Fever * Night sweats		488	1.05	0.63-1.76	0.850

[a] At least one of these comorbidities: HIV infection, diabetes mellitus, liver failure or cirrhosis, congestive heart failure and chronic respiratory disease. CI - confidence interval
