# Supplementary material for: A Prediction Rule to Stratify Mortality Risk of Patients with Pulmonary Tuberculosis
Source: PLoS One. 2016 Sep 16;11(9):e0162797. doi: 10.1371/journal.pone.0162797 (PMC5026366; doi:10.1371/journal.pone.0162797)
Supplement: S2 Table — The marginal effects for each of the predicting variables were compared between both models. The maximum absolute difference found between the two models was 5% (e.g. for patients with age ≥50 years the probability of death within 6 months of diagnosis was 24% with CPR model and 29% with Heckman’s model). However, the CPR model correctly identified the marginal effect associated to each predictor (e.g. when binary variable hypoxemic respiratory failure changes from 0 to 1, the death probability changes 0.23 for CPR and 0.22 with Heckman’s model). Thus, the use of this model showed that missing information in the univariate significant variables had little or no effect on mortality risk assessment. (PDF) [file pone.0162797.s005.pdf]

S2 Table.

Comparison of the marginal effects between the Clinical Prediction Rule (CPR) and the two-stage Heckman model

Predictor	Marginal effects from the CPR (95% CI)	Marginal effects from the 2-stage Heckman model (95% CI)	Marginal effect absolute differences
Hypoxemic respiratory failure			
yes	0.36 (0.27-0-44)	0.40 (0.29-0.51)	0.04
no	0.13 (0.09-0.16)	0.18 (0.14-0.22)	0.05
difference	0.23	0.22	0.01
Age ≥50 years old			
yes	0.24 (0.19-0.29)	0.29 (0.23-0.35)	0.05
no	0.12 (0.08-0.15)	0.16 (0.12-0.21)	0.04
difference	0.12	0.13	0.01
Bilateral lung involvement			
yes	0.22 (0.18-0.25)	0.25 (0.20-0.29)	0.03
no	0.12 (0.07-0.16)	0.17 (0.12-0.22)	0.05
difference	0.10	0.08	0.02
At least 1 significant comorbidity[a]			
yes	0.22 (0.18-0.27)	0.26 (0.21-0.31)	0.04
no	0.13 (0.09-0.17)	0.19 (0.13-0.24)	0.06
difference	0.09	0.07	0.02
Hemoglobin <12 g/dL			
yes	0.21 (0.17-0.25)	0.26 (0.21-0.32)	0.05
no	0.14 (0.10-0.18)	0.18 (0.13-0.22)	0.04
difference	0.07	0.08	0.01

[a] At least one of these comorbidities: HIV infection, diabetes mellitus, liver failure or cirrhosis, congestive heart failure and chronic respiratory disease. CI - confidence interval
