# Supplementary material for: A Prediction Rule to Stratify Mortality Risk of Patients with Pulmonary Tuberculosis
Source: PLoS One. 2016 Sep 16;11(9):e0162797. doi: 10.1371/journal.pone.0162797 (PMC5026366; doi:10.1371/journal.pone.0162797)
Supplement: S3 Table — (PDF) [file pone.0162797.s006.pdf]

S3 Table.

Comparison of the distribution of predictors for derivation and validation cohorts

Predictor	Derivation cohort (n=539)	Validation cohort (n=103)	Total sample (n=642)
Hypoxemic respiratory failure	106 (19.7%)	15 (14.6%)	121 (18.8%)
Age ≥50 years old	241 (44.7%)	43 (41.7%)	284 (44.2%)
Bilateral lung involvement	313 (58.1%)	52 (50.5%)	365 (56.9%)
At least 1 significant comorbidity[a]	252 (46.8%)	47 (45.6%)	299 (46.6%)
Hemoglobin <12 g/dL	259 (48.1%)	37 (35.9%)	296 (46.1%)

[a] At least one of these comorbidities: HIV infection, diabetes mellitus, liver failure or cirrhosis, congestive heart failure and chronic respiratory disease. CI - confidence interval
